# Evolutionary Advantages of Stimulus-Driven EEG Phase Transitions in the Upper Cortical Layers

**DOI:** 10.3389/fnsys.2021.784404

**Published:** 2021-12-08

**Authors:** Robert Kozma, Bernard J. Baars, Natalie Geld

**Affiliations:** ^1^Center for Large-Scale Intelligent Optimization and Networks, Department of Mathematics, University of Memphis, Memphis, TN, United States; ^2^Center for the Future Mind, Florida Atlantic University, Boca Raton, FL, United States; ^3^Society for MindBrain Sciences, San Diego, CA, United States; ^4^MedNeuro, Inc., New York, NY, United States

**Keywords:** machine understanding, cortex, perception, consciousness, graph theory, neuropercolation, phase transition, criticality

## Abstract

Spatio-temporal brain activity monitored by EEG recordings in humans and other mammals has identified beta/gamma oscillations (20–80 Hz), which are self-organized into spatio-temporal structures recurring at theta/alpha rates (4–12 Hz). These structures have statistically significant correlations with sensory stimuli and reinforcement contingencies perceived by the subject. The repeated collapse of self-organized structures at theta/alpha rates generates laterally propagating phase gradients (phase cones), ignited at some specific location of the cortical sheet. Phase cones have been interpreted as neural signatures of transient perceptual experiences according to the cinematic theory of brain dynamics. The rapid expansion of essentially isotropic phase cones is consistent with the propagation of perceptual broadcasts postulated by Global Workspace Theory (GWT). What is the evolutionary advantage of brains operating with repeatedly collapsing dynamics? This question is answered using thermodynamic concepts. According to neuropercolation theory, waking brains are described as non-equilibrium thermodynamic systems operating at the edge of criticality, undergoing repeated phase transitions. This work analyzes the role of long-range axonal connections and metabolic processes in the regulation of critical brain dynamics. Historically, the near 10 Hz domain has been associated with conscious sensory integration, cortical “ignitions” linked to conscious visual perception, and conscious experiences. We can therefore combine a very large body of experimental evidence and theory, including graph theory, neuropercolation, and GWT. This cortical operating style may optimize a tradeoff between rapid adaptation to novelty vs. stable and widespread self-organization, therefore resulting in significant Darwinian benefits.

## 1. Introduction

### 1.1. Computers, Brains, and Energy

We tend to think of the field of computers and informatics as a major event in the history of ideas, and that is broadly correct. But the mathematics of computation can be traced back to ideas propounded by philosophers and linguists at least a thousand years ago. Western and Asian traditions are often traced to the first millennium BCE; certainly the readable scripts of that time seem to reveal ideas and observations that are remarkably “modern.” History is itself a massively parallel distributed network of events over many centuries. It was not until the invention of digital computers about 80 years ago that systematic studies became feasible to explore the possibility of developing man-made intelligent machines (Turing and Haugeland, 1950; Von Neumann, 1958), which have the potential of demonstrating problem-solving performance comparable to humans. Computer technology demonstrated exponential growth for over half a century. Computers support all aspects of our life. Indispensable and pervasive, they lift billions of people out of poverty worldwide and help them to benefit from technological progress in a modern, interconnected society. The dominant approaches in these applications use Neural Networks (NNs) (Barto et al., 1983; Bishop, 1995; Miller et al., 1995) and Deep Learning (DL), and produce cutting-edge AI with often super-human performance (LeCun et al., 2015; Mnih et al., 2015; Schmidhuber, 2015). The present development trend of intelligent technologies is unsustainable. DL has very high demand for computational power and it requires huge data resources, raising many questions from engineering, societal, and ethical perspectives (Jordan and Mitchell, 2015; Marcus, 2018; Kozma et al., 2019a). Computer chips reach hard limits, marked by the approaching end of Moore's law, which dominated computer development for over half a century (Waldrop, 2016). Energy considerations are an important part of the challenges. High-performance computers require increasing proportions of the available electrical energy to operate (Amodei et al., 2018). Moreover, it is increasingly complicated to remove the heat dissipated in the densely packed microchip circuitries.

Brains provide us valuable clues regarding efficient use of resources, including energy. The operation of brains is naturally constrained by the available metabolic resources following fundamental laws of thermodynamics. According to the free energy principle, brains optimize metabolic and computational efficiency by reconfiguring themselves while they interact with the environment in the action and perception cycle (Friston et al., 2006; Sengupta et al., 2013). Brains continuously optimize their energy resource allocation, while advanced computing algorithms are mostly agnostic when it comes to power consumption. Arguably, brains are several orders of magnitude more energy-efficient than cutting-edge AI when solving specific machine learning tasks (Amodei et al., 2018; Kozma et al., 2019b; Marković et al., 2020). The efforts to achieve human-level intelligence and machine understanding by scaling up computing using million-core chips are impressive, but alternative approaches may become useful as well. Energy-awareness is a basic manifestation of embodiment, which is crucial for the emergence of intelligence in brains and machines (Dreyfus, 2007), and it provides the key for progress in machine understanding as well (Yufik, 2013, 2019). Neuromorphic technologies have great potential in large-scale computing systems, including spiking neural networks (Furber, 2016; Hazan et al., 2018; Roy et al., 2019), and memristive hardware (Di Ventra et al., 2009; Chua, 2012; Kozma et al., 2012; Stieg et al., 2019). Combining neuromorphic technologies with brain-inspired thermodynamic models of computing has the potential of providing the required breakthrough in machine understanding (Yufik and Friston, 2016; Friston et al., 2020).

### 1.2. Cognitive Dynamics and Consciousness

It is often thought that the question of consciousness in the waking brain is so difficult and poorly understood that empirical science has nothing to say about it. However, beginning some decades ago, empirical scientists in psychology and neuroscience have published literally thousands of scientific papers, mostly on very specific aspects of conscious perception and cognition.^1^ Global Workspace Theory (GWT) is one of the prominent modeling approaches (Baars, 1997; Baars and Geld, 2019; Baars et al., 2021). GWT fundamentally proposes that the striking capacity limits of conscious percepts implies very widespread unconscious access to processing resources in the brain. This convergence of two very different theoretical traditions suggests that they are two sides of the same coin.

GWT first emerged around 1980, based on the cognitive architecture tradition in cognitive science, including global workspace architecture (Newell et al., 1972). The cognitive architecture program goes back many decades, when Herbert A. Simon and the Netherlands chess master Adrian De Groot began to carefully study the move-by-move “consciousness reports” of advanced chess players (Simon, 1967; De Groot, 2014). Since the middle of the last century, a number of cognitive architectures have been proposed and partially tested. The book by Newell (1994) can be considered to be a summary of this empirical modeling tradition. At least a dozen cognitive architectures have been proposed in this research practice. They proposed different computer implementations with two shared features: All cognitive architectures had a serial perception and problem-solving component, and in all cases the serial flow of immediately accessible events interacted with a very large long-term memory capacity, which appears to be a non-serial set of knowledge sources. Cognitive architectures also merged with a separate experimental cognitive research tradition, until, by the 1970s and 80s, it began to seem that both lines of research could be understood in a single framework (John and Newell, 1990). The work of Tversky and Kahneman (2011) is another example of this pattern of discoveries, focusing on the empirical phenomenon of automaticity. Newell (1994) discussed this striking convergence of a serial “stream of consciousness” reported by subjects, and a very large, non-serial set of memory domains, which are not in reportable consciousness at any given time; but the massively parallel memory domain is unconscious most of the time during chess playing.

Baars was one of the first cognitive scientists to explicitly use the word “conscious” for the serial component of chess-playing protocols, and “unconscious” for the large set of knowledge sources that players demonstrably use, but which may not become explicit in any single chess move. What Baars called Global Workspace Theory (GWT) in the 1980s combined two streams of scientific study, the cognitive architecture tradition and the field of cognitive psychology (Baars, 1997). That convergence seemed to be surprisingly easy to describe. By 1980 the field of cognitive science began to emerge, and the computational, mathematical, and cognitive-behavioral streams of development turned into a single, extensive field of study. Baars' GWT linked a vast empirical literature to the theoretical concept of consciousness, which could be inferred from the mass of evidence, and which also seemed to reflect the reported experiences of subjects in many tasks.

The distinctive feature of all cognitive architectures, including GWT, can be found in Newell's pioneering formulation. Rather than a passive unconscious long-term memory, with more powerful computers the idea emerged that the parallel component reflects a “society” of specialized knowledge sources that were not conscious by themselves, but which interacted to “post messages” on some shared knowledge domain, called a global workspace. Since that time, computational GWT has seen very widespread use in cognitive and computer science. The mathematics of parallel-interactive computation led to both fundamental and practical insights into human cognition. What seemed puzzling and scattered before 1980 gradually emerged with a greater degree of clarity (Franklin et al., 2012).^2^

Cognitive Science is now Cognitive Neuroscience, leading to another large set of converging ideas, with more and more brain and behavioral evidence interacting in fruitful ways. In fields like language studies, for example, it became routine to consider the perceptual aspects of a stream of words (like this one) as conscious, in fast-cycling interaction with multiple unconscious knowledge domains. “Society models” gradually merged with the brain sciences, giving rise to contemporary cognitive neuroscience theory. We prefer to think of a “family” of GWT architectures, where Baars' version is perhaps the best known today, but the family has many members that continue to evolve. Essentially empirical, this set of theories may be considered similar enough to be treated as a “family” of global workspace-like approaches, including (Dehaene et al., 1998; Fingelkurts et al., 2010; Edelman et al., 2011; Tononi and Koch, 2015; Kozma and Freeman, 2016; Mashour et al., 2020; Deco et al., 2021). Each approach is distinctive and each is based on a strong body of evidence; but they converge well. Much to our surprise, a very large scientific literature in neurobiology has also converged with all the fields in a remarkable way.

The current paper presents yet another region of convergence between multiple empirical and theoretical streams of development. With direct brain recordings of the electromagnetic activity of single neurons and massive neuronal networks, we may be seeing a convergence between many intellectual traditions. We view brains as large-scale complex networks, and brain dynamics as percolation processes evolving over these networks, with potentially adaptive structures. We introduce several key analysis methods, such as the thermodynamics of wave packets, statistical physics of criticality and phase transitions, cinematic theory of neurodynamics and metastability, and a hypothesis concerning the interpretation of the experimentally observed neurodynamics using the GWT framework. Two main computational results are introduced to illustrate the findings. The first describes the essential role of non-local axonal connections in maintaining a near-critical state of brain oscillations. The second result concerns the role of astrocyte-neural coupling in maintaining neural fields with rapid transitions between states with high and low synchrony, respectively. We conclude the work with discussing the potential implications of these results to lay down the principles of machine understanding.

The rest of the essay addresses the fundamental question: What could be the evolutionary advantage of brains utilizing phase transitions, as compared to possible alternatives with smooth dynamics?

## 2. Methods

Describing brains as open thermodynamic systems converting noisy sensory inputs and metabolic energy into conscious sensory percepts to explicit understanding of the world.

### 2.1. Thermodynamics of Wave Packets^3^

There is a vast literature on experimental investigations of thermodynamics of brains, see, e.g., Abeles and Gerstein (1988); Fuchs et al. (1992); Freeman (2000), and Friston et al. (2006). Freeman K sets provide a theoretical framework for brain models with a hierarchy of increasingly complex structure, dynamics, and function (Freeman, 1975, 1991, 2000; Kozma and Freeman, 2009). Several key aspects are summarized here, using the concept of metastability,^4^ as described in Kozma and Freeman (2016, 2017).

**P****ROPOSITION 1** (Characterization of wave packets (WPs); Kozma and Freeman, 2016). *The action-perception cycle is manifested through the self-organized sequence of metastable, highly synchronized patterns of spatio-temporal amplitude modulated (AM) activity at the beta/gamma carrier frequency (20-80 Hz). These AM patterns emerge and collapse, and as such they form spatio-temporal Wave Packets (WPs). The WPs evolve as follows:*

*(i) WPs exist for a time window of* ~*100 ms, corresponding to approx. 10Hz frequency band. They have spatially-localized evolving patterns, therefore they are sometimes called wave packets*.
*(ii) WPs have statistically significant correlations with sensory stimuli and reinforcement contingencies perceived by the subject*.
*(iii) WPs are separated in time by brief transitionary periods (10-20ms). During these transitionary periods, the AM patterns collapse and large-scale synchrony diminishes*.
*(iv) The repeated collapse of WPs points to recurring singularities in mammalian cortical dynamics ignited at a given location of the cortex. Following the selection and activation of a Hebbian cell assembly corresponding to the stimulus, the synchronized activity of neural populations rapidly propagates across the cortical sheet in the form of a phase cone*.
*(v) The rapid transitions and propagation of phase cones following their ignition cannot be explained by synaptic transmissions only, and it requires the emergence of collective dynamics*.

The repeated collapse and emergence of the metastable wave packets defines a quasi-periodic oscillatory energy cycle with the following steps:

**P****ROPOSITION 2** (Energy cycle of wave packets; Kozma and Freeman, 2017). *The temporal evolution of Wave Packets is sustained by the corresponding energy cycle, described by thermodynamic processes involving energy and entropy transfer between highly-ordered (liquid) states and disordered (gaseous) states:*

*(i) The cycle starts with a disordered background state with low amplitude waves. This state has high entropy and in the thermodynamic sense it is analogous to a gaseous state*.
*(ii) At a certain space-time point, synchrony is ignited in the neural populations in response to a meaningful stimulus and a phase cone starts to grow from an incipient state. The phase cone develops into a highly structured, metastable WP with low entropy oscillating at a narrow beta/gamma frequency band. The emergence of the WP leads to the dissipation of energy in the form of heat, which is removed by the blood stream through the capillaries. This can be viewed as a condensation process to a liquid state*.
*(iii) The metastable WP continuously erodes with decreasing synchrony between the neuron components, due to the impact of input stimuli and random perturbations. The entropy increases, which corresponds to the thermodynamic process of evaporation*.
*(iv) At the end of the cycle, the intensity of the neural firing activity drops to a level when the activity patterns are dissolved and the thermodynamics returns to the high-entropy gaseous state*.

This section summarized key aspects of experimental findings on EEG recordings in terms of thermodynamic processes. The next sections introduce methods of statistical physics and mathematical theory of graphs and networks to quantitatively characterize these findings.

### 2.2. Criticality in Brains and Neuropercolation Model

The thermodynamic interpretation of the action-perception cycle outlined above implies that brains operate through repeated transitions between highly-organized, synchronous states and disorganized states with low levels of synchrony. These observations lead to the hypothesis that brains are critical or near-critical systems, which has been proposed by various authors. One prominent approach is based on the concept of self-organized-criticality (SOC) when a high-dimensional complex system organizes itself to a critical point which is an attractor state. SOC demonstrates scale invariance, including power-law behavior with 1/*f* scaling, where *f* is the frequency of the events corresponding to the specific problem domains. In the case of neural processes, *f* could relate, for example, to bursts of spontaneous activity in neural populations, and 1/*f* shows the number of bursts of the given frequency. SOC has been observed in many disciplines, from earthquakes, to solar flares, sandpiles, etc, and in neural tissues as well (Beggs and Timme, 2012; Shew and Plenz, 2013). SOC is widely used now in the interpretation of brain monitoring data, including the connectome, resting state networks, consciousness, and other areas; see, e.g., Fingelkurts et al. (2013); Tagliazucchi (2017); Nosonovsky and Roy (2020), and Wang et al. (2020). Under certain conditions, deviation from the power-law behavior predicted by SOC are observed in brain dynamics, which justify approaches addressing criticality beyond SOC, e.g., critical integration and soft assemblies (Aguilera and Di Paolo, 2021).

A related approach uses percolation theory to describe criticality of brain operation, by modeling the cortical neuropil (Kozma et al., 2005, 2014; Bollobás et al., 2010; Kozma and Puljic, 2015).

**P****ROPOSITION 3** (Neuropercolation model of criticality and phase transition in brain dynamics; Kozma et al., 2005; Kozma and Puljic, 2015). *According to neuropercolation, critical behavior in the cortex is made possible by the filamentous structure of the cortical neuropil, which is the most complex substance in the known universe. Neuropercolation is the generalization of Ising models and lattice cellular automata, and it describes the following aspects of the neuropil:*

*(i) Presence of rare long axonal connections between neurons, which allow action at distant locations with minimal delay*.
*(ii) Contribution of astrocytes cells, which have a key role in metabolic processes and in the formation of field effects*.
*(iii) Incorporation of random noise effects; the model is robust to noise and noise is an important constructive control parameter to tune the system to achieve desired behavior*.
*(iv) Input-induced and spontaneous phase transitions between states with large-scale synchrony and without synchrony exhibit brief episodes with long-range spatial correlations*.
*(v) Neuropercolation proposes a constructive algorithm that self-regulates cortical dynamics at criticality following supercritical explosive excursions*.

Beyond the theoretical results, neuropercolation has been employed successfully to interpret experiments with Pavlovian conditioning in rabbits (Kozma et al., 2014; Kozma and Puljic, 2015), on entrainment of sensory processing by respiration in rats and human subjects (Heck et al., 2017, 2019), and strategy changes during learning in gerbils (Kozma et al., 2021).

### 2.3. Intermittent Metastable Brain Oscillations

There is widespread agreement that processing of sensory information in the cortex is associated with complex spatio-temporal patterns of activity (Abeles, 1982). Experimental observations of intermittent brain oscillations with extended metastable periods, interrupted by rapid transients, are widely discussed in the literature (Lehmann et al., 1987; Buzsáki, 1998). This issue is often framed as a choice between opposing views of continuous vs. discrete cognition. Following the wisdom of Kelso's complementarity principle, the likely answer would be that both discrete and continuous aspects are relevant to cognition through the unity of continuity-discreteness (Fingelkurts and Fingelkurts, 2006; Tognoli and Kelso, 2014; Parr and Friston, 2018). Recent reviews by Josipovic (2019), Menétrey et al. (2021), and Lundqvist and Wutz (2021) help to disentangle the arguments.

The hypothesis that perception happens in discrete epochs has been around for decades, and models of brains as dynamical systems with itinerant trajectories over distributed attractor landscapes provided mathematical tools to support the analysis, see, e.g., Babloyantz and Destexhe (1986); Skarda and Freeman (1987); Freeman (2000), and Tsuda (2001). Crick and Koch (2003) described discrete frames as snapshots in visual processing, as well as in consciousness; while Tetko and Villa (2001) provided evidence of cognitive relevance of spatio-temporal neural activity patterns. The sample-and-hold hypothesis expands on the sampling idea and it describes the perceptual and motor processing cycle (Edelman and Moyal, 2017). Spatiotemporal sequences of time-position patterns have been observed in the human brain associated with cognitive tasks (Tal and Abeles, 2018). Recent models describing sequential processing of complex patterns of brain activity are developed in, e.g., Cabessa and Villa (2018); Malagarriga et al. (2019).

EEG data evaluated using Hilbert analysis also display sudden transitions of cognitive relevance (Brennan et al., 2011; Frohlich et al., 2015), while operational architectonics provides a powerful framework for transient synchronization of operational modules underlying mental states (Fingelkurts et al., 2010, 2017). Phase transitions over large-scale brain networks have been applied to describe the switches from one frame to another in the cinematic theory of neurodynamics and cognition (Kozma and Freeman, 2016, 2017). Kozunov et al. (2018) evaluates MEG visual processing data and points to the role of phase transitions and critical phenomena to understand how meaning can emerge from sensory data. The identified cycle length varies depending on the experimental conditions; i.e., it is in the theta/bands in the cinematic theory (Freeman, 2000; Kozma and Freeman, 2017); while Pereira et al. (2017) estimate a very long cycle of consciousness (2 s). The work by Werbos and Davis (2016) is unique by identifying a very precise clock cycle of 153 ms, by analyzing Buzsáki lab data (Fujisawa et al., 2015).

There are various open issues regarding discrete effects in neurodynamics and some questions were raised about their significance in cognition and consciousness. For example, Fekete et al. (2018) states that the involved brain networks cannot produce switching behavior at the rates observed in brain imaging experiments. They lay out a valuable work, but they do admit that their reasoning does not hold for strongly non-linear systems as brains are. Their proposed multi-scale computation near criticality is certainly interesting and it has a lot in common with the edge of criticality described as the result of ontogenetic development in neuropercolation in the past two decades (Kozma et al., 2005). White (2018) does not question the existence of sudden changes observed by Freeman et al. (2006); Brennan et al. (2011), and Kozma and Freeman (2016), rather it misses the established proof that these neurodynamic effects are relevant to conscious perception. Clearly, there is a need for extensive further experiments before confirming or rejecting the central hypothesis on the key role of phase transitions in cognition and consciousness. Some recent experiments lend support to the hypothesis on discontinuities in cognition, such as entrainment of multi-sensory perception by the respiratory cycle (Heck et al., 2017); how breathing shapes memory functions (Heck et al., 2019); the role of state transitions in strategy changes during an aversive learning paradigm and the formation of Hebbian cell assemblies by identifying emergent causal cortical networks (Kozma et al., 2021); and clustering of phase cones during interictal periods over the epileptogenetic brain region (Ramon and Holmes, 2020). Statistical markers of phase transitions show potential use in psychotherapy (Sulis, 2021).

**P****ROPOSITION 4** (Transient processing in perception; Kozma and Freeman, 2017). *Phase transitions over large-scale brain networks have been applied to describe the switches from one frame to another in the cinematic theory perception, as follows:*

*(i) The intermittent emergence and collapse of AM patterns in EEG data is interpreted as the evidence that perceptual information processing happens in discrete steps, aligned with the prominent AM patterns*.
*(ii) The cinematic theory of perception uses the concept of the frame and the shutter, which follow each other sequentially. There is no exact threshold separating the two phases from each other, rather they transit to each other following the corresponding energy cycle of WP*.
*(iii) The frames are defined by the dominant AM patterns which are sustained for an extended period of around 100 ms, with significant variation depending on experimental conditions. The frames are selected according to the reinforced contingencies as perceived by the subject. The frame activity is largely synchronous across large cortical areas during the existence of the frame. However, the frame is not a frozen pattern, rather it oscillates at the beta/gamma carrier frequencies*.
*(iv) The shutter is defined by the relatively short periods (approx. 10 ms) when the AM patterns collapsed and the neural activity is disordered, still not completely random and maintains some trace of the previous dynamics*.

The Freeman/Kozma approach has been called *cinematic*, because the cortical dynamics self organizes into phase plateaus at roughly every ~ 100 ms, followed by a collapse of the phase plateau for about 10 ms. During the brief collapse of synchrony, the cortex is prepared to receive novel perturbations, while the self organized phase synchrony is a time of relative stability and internal processing. This style of functioning plausibly optimizes a balance between receptivity to novelty and stability, pointing to potential evolutionary advantage by the rapid, moment-to-moment adaptivity of the conscious cortex.

Brains are dynamic systems, they can never stop, not even during the relatively quiet periods when frames with metastable amplitude patterns are maintained. Being constrained to a quasi-periodic attractor basin during a frame is just the sign of relative silence, before the explosive impact of the phase transition, which destroys the existing structure and gives rise to the emergence of a new pattern in response to the new sensory input and its meaning to the subject (Freeman, 2000). Dynamical modeling of the brain includes both continuity of the movement along its trajectory, as well as rapid changes as the path leads from one metastable state to another (Tognoli et al., 2018). The switches are not rigid and they have their own rich dynamic structure and a hierarchy with possibly scale-free distribution (Mora-Sánchez et al., 2019). These results show that an integrative approach to identify major features of cognitive dynamics and consciousness is very productive, including the unity of discrete and continuous operating modalities in brains.

### 2.4. Hypothesis on the Link Between EEG Perceptual Transition and GWT

Phase transitions and criticality in cortical layers may have a profound impact on the nature of consciousness. There have been various attempts to integrate phase transitions with GWT, such as the one by Werner (2013), to model the emergence of multi-level collective behaviors in brain dynamics. Tagliazucchi (2017) describes consciousness as the integration of fragmented, highly differentiated entities into a unified message, and they use percolation model to describe the propagation of conscious access through the brain network medium, with phase transitions when a critical threshold is reached. Josipovic (2019) elaborates the concept of non-dual awareness in the framework of GWT. GWT is hereby linked to perceptual phase transitions (Freeman, 1991, 2000; Kozma and Freeman, 2016).

**P****ROPOSITION 5** (Main Hypothesis on EEG phase transitions as indications of conscious experience Kozma and Freeman, 2016; Baars and Geld, 2019). *Phase transitions in the cortex are ignited at a given location of the cortex, according to EEG data. Phase transitions generate laterally propagating phase gradients (phase cones) across the cortical sheet. In the context of GWT, these results are interpreted as follows:*

*(i) Phase cones are neural signatures of perceptual broadcasts described by GWT*.
*(ii) The rapid expansion of phase cones, covering large cortical areas within 10-20 ms, are consistent with the propagation of perceptual broadcast postulated by GWT*.
*(iii) The recurrence time of the cortical phase transitions is about 100 ms, which is consistent with the* ~ *100 ms window identified in numerous perceptual and behavioral experiments*.

The ~ 100 ms time domain has long been studied in the sensory sciences and proposed as an integration period for conscious cortical information processing (Baars, 1988; Madl et al., 2011; Baars and Geld, 2019). GWT suggests that conscious sensory events are the leading edge of adaptation during waking life. The very fast and highly adaptive role of cortex clearly fits within a Darwinian framework of genetic, epigenetic, and moment-to-moment cortical adaptation (Edelman et al., 2011). Edelman's Neural Darwinism is highly consistent with this approach, and specifies the role of selectionism at multiple time and spatial scales in the brain. Interpreting phase cones as neural manifestations of perceptual broadcasts of GWT is an important step to connect the content of consciousness with the temporal structure of consciousness *per se* (Menétrey et al., 2021). Next, computational results are introduced to illustrate the hypothesis.

## 3. Results

### 3.1. Long-Axonal Connections Facilitate Criticality in the Neuropil

Brain networks analysis has been successful to study anatomical, functional, and effective brain connectivity, using tools of graph theory (Iglesias and Villa, 2007, 2010; Stam and Reijneveld, 2007; Steyn-Ross and Steyn-Ross, 2010; Bullmore and Sporns, 2012; Haimovici et al., 2013). Imamoglu et al. (2012) suggest that frontal and visual brain regions are part of a functional network that supports conscious object recognition by changes in functional connectivity. Zanin et al. (2021) point out that neuroscience of brain networks often emphasizes the extraction of neural connectivity represented by strong links and highly-connected nodes, although weak links can in fact be critical in determining the transition between universality classes. Most of the existing network-based toolsets extract information on the interaction of localized units and nodes (Korhonen et al., 2021). Brains are metastable systems, and their optimal functioning depends upon a delicate metastable balance between local specialized processes and their global integration (Fingelkurts and Fingelkurts, 2010), while minute perturbations and topological changes can lead to significant deviations from the normal operational dynamics (Tozzi et al., 2017), with an impact on synchronization effects in these complex non-linear systems (Brama et al., 2015; Xu et al., 2020). Random graphs and cellular automata models have been developed for cortical dynamics to address the challenges (Balister et al., 2006; Kozma and Puljic, 2015; Ajazi et al., 2019; Turkheimer et al., 2019). Percolation models are especially helpful in the interpretation of experimental findings describing the intermittent emergence of common-mode oscillations in neural cell assemblies (Kozma and Freeman, 2016).

An important theoretical finding describes phase transitions in a graph model of the cortical neuropil with a mix of short and long connections, including long axons (Janson et al., 2019). A random graph $G_{{\mathbb{Z}}_{N}^{2},p}$ is considered over the square grid of size (*N*+1) × (*N*+1), and *p* is the probability describing random long edges, see Equation (1). We assume periodic boundary conditions, for simplicity, thus we have a torus with the short notation ${\mathbb{Z}}_{N}^{2}$. The set of vertices of *G* consists of all the vertices of ${\mathbb{Z}}_{N}^{2}$. There are two types of edges *E*, short and long, respectively. Short edges are all the edges from the torus ${\mathbb{Z}}_{N}^{2}$; i.e., each node has 4 short edges connecting to its 4 direct neighbors. Additionally, we introduce random long edges as follows: for any pair of vertices that are at distance *d* apart of each other on the lattice, we assign an edge with probability *p* that depends on the distance:


(1) $$\begin{array}{l} p_{d}=\mathbb{P}\left(\left(x,y\right)\in E\left(G_{{\mathbb{Z}}_{N}^{2},p}\right)\text{ and }dist\left(x,y\right)=d\right)=c/\left[Nd^{\alpha }\right], \end{array}$$

Here α is a number, e.g., α = 1. An activation process is defined on $G_{{\mathbb{Z}}_{N}^{2},p}$ as follows: Denote by *A*(*t*) the set of all active vertices at time *t*. We say that a vertex *v* is active at time *t* if its potential function χ_*v*_(*t*) = 1 and inactive if χ_*v*_(*t*) = 0. Therefore, *A*(*t*) = {*v*∈*V*(*G*) |χ _*v*_(*t*) = 1}. At the start, *A*(0) consists of all vertices that are active with probability *p*_0_. Each vertex may change its potential based on the states of its neighbors as follows:


(2) $$\begin{array}{l} \chi_{v}\left(t+1\right)=1\left(\underset{u\in N\left(v\right)}{\sum }\chi_{u}\left(t\right)\geq k\right) \end{array}$$

A vertex can become active if at least *k* of its neighbors are active. Let ρ_*t*_ be a proportion of active nodes at time *t*, i.e., $\rho_{t}=A\left(t\right)/N^{2}$ then the evolution of ρ_*t*_ can be described in a mean-field approximation, for details, see Janson et al. (2019). A key result has been derived for the existence of phase transition of the activation process over $G_{{\mathbb{Z}}_{N}^{2},p}$:

**P****ROPOSITION 6** (MAIN THEOREM JKRS219: on phase transitions in the neuropercolation model with short and long connections Janson et al., 2019). *For the activation process* *A*(*t*) *over random graph* $G_{{\mathbb{Z}}_{N}^{2},p_{d}}$*, in the mean-field approximation, there exists a critical probability* *p*_*c*_ *such that for a fixed* *p, w.h.p.:*

1. *all vertices will eventually be active if* *p*>*p*_*c*_*, while*
2. *all vertices will eventually be inactive for* *p*<*p*_*c*_.
3. *The value of* *p*_*c*_ *is given as the function of* *k* *and* λ *through the solution of some transcendental equations*.

**Figure 1:**
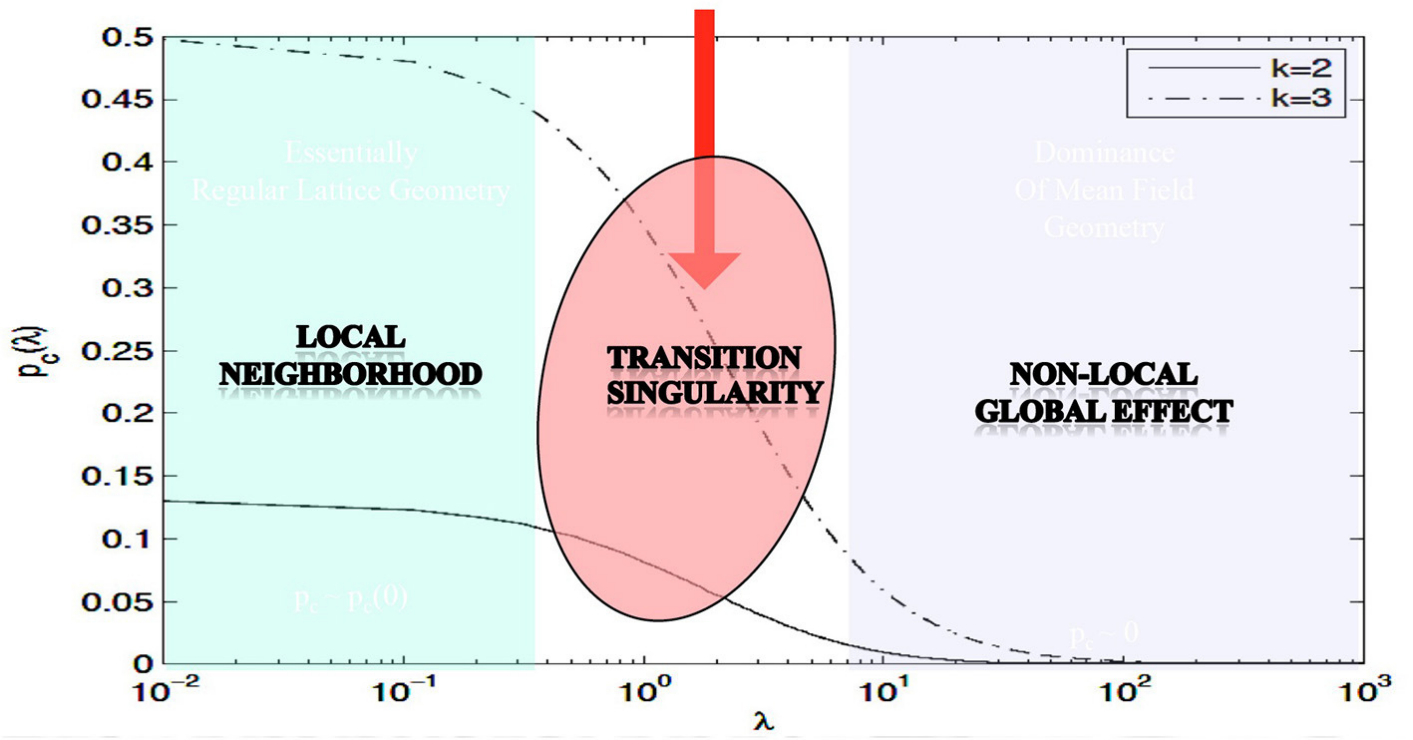
Illustration of the effect of the long edges λ on the critical probability *p*_*c*_; parameter *k* specifies the type of the update rule; based on Janson et al. (2019).

The main theorem in Proposition 3.1 rigorously proves the existence of phase transitions in neuropercolation model with long axons; its meaning is illustrated in Figure 1, using numerical evaluation of the precise mathematical formula. In Figure 1, the x-axis shows λ, which scales linearly with the probability of long axons, while the y-axis is the critical probability when the phase transition happens; *k* indicates the update rule. It is seen that there is a region for small λ values, where the model behaves essentially as a *local system*. For large λ values, the critical probability diminishes what is expected for a *global system* without local order. There is a transitionary region when the incremental addition of long connections does matter, as it is expected to be the case in the neuropil. Clearly, this model cannot grasp all the complexity of brain networks, and there are many advancements including inhibitory and excitatory effects, multi-layer architectures with delayed reentrant connections. Still, the introduced effect is very robust and it is a unique property of the neuropil with a mix of short and long projections. Brains can benefit from the transitionary region for tuning their behavior between local fragmentation and overall global dominance, using adaptation and learning effects.

### 3.2. Metabolic Processing in the Neuropil Controls Transitions Between States With High and Low Synchrony Based on Hysteresis Dynamics

Following fundamental studies on the brain energy budget (Raichle and Gusnard, 2002; Magistretti, 2006), there are extensive integrative models on metabolic coupling in the neuron-glia ensemble with capillaries (Cloutier et al., 2009; Belanger et al., 2011; Jolivet et al., 2015), and the role of metabolic constraints on spiking activity (Teixeira and Murray, 2015; Zhu et al., 2018; Qian et al., 2019). The models typically use multi-compartmental neuron models, but some simplified still realistic spiking neuron models are popular as well, e.g., Izhikevich (2003).

To describe the emergence of synchronized collective cortical oscillations driven by metabolic constraints, the capillary astrocyte-neuron model (CAN) is introduced, which couples spiking and metabolic processes (Kozma et al., 2018, 2019b). The simplest CAN model has two metabolic variables: *g*(*t*) and *m*(*t*), where *g*(*t*) describes the available glycogen stored in the astrocyte, *m*(*t*) models the available ATP in the neuron's mitochondria. Izhikevich (2003) model is used for the spiking neurons, with variables *u*(*t*) and *v*(*t*), which are the dimensionless membrane potential and the membrane recovery variable, respectively. The following differential equations describe the rate of change of the variables:


(3a) $$\begin{array}{ll} dv/dt & =\;\Phi_{1}\left(u,v\right)+I\left(t\right)\\ du/dt & =\;\Phi_{2}\left(u,v,b^{+}\left(m\right)\right) \end{array}$$

(3b) $$\begin{array}{ll} dg/dt & =-{\text{Ψ}}_{1}\left(g,m\right)+\kappa \int_{t-\tau }^{t}v\left(t^{\prime }\right)dt^{\prime }\\ dm/dt & =-{\text{Ψ}}_{2}\left(g,m\right)+{\text{Ψ}}_{1}\left(g,m\right) \end{array}$$

Here Φ_1_(*u, v*) is membrane potential fitting function; Φ_2_(*u, v, m*) describes the recovery variable dynamics, modulated by the available ATP via *m*(*t*). Ψ_1_(*g, m*) and Ψ_2_(*g, m*) describe the attenuation of *g*(*t*) and *m*(*t*), respectively. *I*(*t*) describes the influence of synaptic currents. The integral term in Equation (3b) describes the cumulative effect of spiking on the glutamate concentration in the synaptic cleft, over time period of τ, and κ is a scaling parameter. Izhikevich's model has a sensitivity parameter *b* regulating spike production inside term Φ_2_(*u, v, m*). A nominal value of *b* = 0.2 assures regular spiking (Izhikevich, 2003). To close the feedback loop between the metabolic and neural parts of the model, *b* is modulated by *m*(*t*) as follows: *b*^+^(*m*(*t*)) = [ω*b*+β*m*(*t*)]. Here ω is a scaling parameter in the range [0.75, 1.25], directly impacting the spiking density. The 2nd term reflects the contribution of *m*(*t*), where β is a control parameter in the range [0, 0.5]. For β = 0, metabolic processes do not impact spiking, while increasing β leads to increasing frequency of spiking. Full elaboration of the model is given in Kozma et al. (2018).

**Figure 2:**
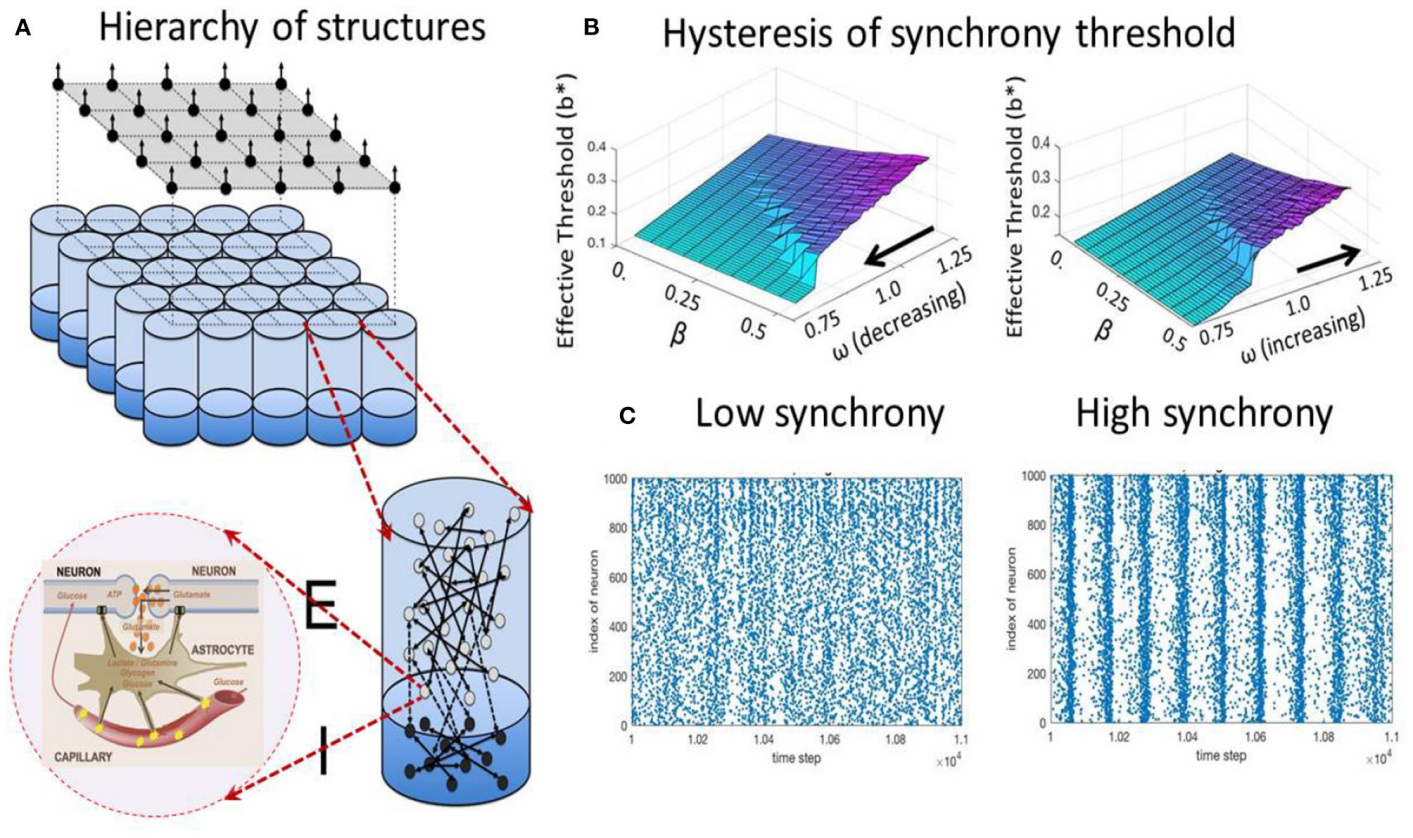
Metabolic-neural model; **(A)** hierarchy of structures from cellular, mini-column, and mesoscopic population levels; **(B)** hysteresis effect on the transition between states with low (blue/turqoise) and high synchrony (purple); **(C)** examples of spiking raster plots for low and high synchrony.

**P****ROPOSITION 7** (Metabolic control of synchrony transitions in neural populations based on hysteresis dynamics Kozma et al., 2019b). *The capillary astrocyte-neuron model (CAN) described by Equations (3a)–(3b) demonstrates transitions between synchronized collective cortical oscillations and the absence of synchrony, as illustrated in* *Figure 2**. The process has the following properties:*

*(i) The amount of available energy modulates the oscillation frequency of neural populations*.
*(ii) There is a hysteresis effect as the result of cusp bifurcation in the CAN model. The space defined by the forward gain from neural to metabolic subsystems, and the feedback gain from metabolic to neural system has a bifurcation point leading to the split of a stable equilibrium to two stable and one unstable equilibrium*.
*(iii) The parameters corresponding to the bifurcated states produce self-sustained oscillations between high and low-synchrony states*.
*(iv) The results reproduce experimentally observed collective neural dynamics in the form of large-scale cortical phase transitions*.

It is important to point out that the metabolic processes are required to produce the hysteresis effect and the desired transitions between states with high and low synchrony. Populations of pure spiking neurons without metabolic components are not sufficient to reproduce the experimentally observed transition effects, as it has been remarked by Deschle et al. (2021).

## 4. Discussion: Human Understanding and Machine Understanding

This work explores what the evolutionary advantage may be of brains utilizing repeated phase transitions at theta/alpha rates, as compared to possible alternatives with smooth dynamics. There are a striking number of regularities that are found over and over again at around 10 Hz. Some of these emerge from the mathematics of neurodynamics described here, and some of them emerge from a century of research in conscious sensory perception. We can call this pattern of convergence the “magic number” near-10 Hz (~100 ms). The flow of conscious events is serial, while unconscious knowledge domains constantly interact with the conscious stream, as EEG data and psychological evidence show over and over again. The ~100 ms Temporal Window has been studied since the 1800s because it keeps on emerging in psychological evidence. In psychology experiments, it is always linked to highly reliable reports of conscious sensory experiences. As we described here, the magic Temporal Window may be explained by the cinematic view of neurodynamics and phase transitions in the cortex. Because the ~100 ms Temporal Window is so common, and clearly appears in association with conscious experiences, this possible link is intriguing.

Some of the empirical phenomena that clearly dwell in the magic Temporal Window:

1. Two sensory inputs fuse into single conscious gestalts if they occur within a ~100 ms time window. This is an enormously general phenomenon in sensory psychophysics, both within and between the major sensory modalities.
2. The motor domain shows a similar Temporal Window. Simple reaction time hovers around ~100 ms. In continuous tasks, the relationship between sensory output and motor outputs works best within the Temporal Window.
3. The ~ 100 ms sensory integration window is found in all the major senses, and also in cross sensory tasks. We should reemphasize the extraordinary generality of this phenomenon across vision, audition, and touch perception in humans and other species. What has been missing is an explanation.

The mathematical properties of cortex, as found by Kozma and Freeman (2016), may therefore explain unconscious-conscious events as they have long been observed in psychology experiments. Phase transitions create the basis for rapid and robust responses to environmental challenges, which provided our ancestors with evolutionary advantage compared to the competitors. As an illustration of these abstract considerations, we can easily imagine a wild rabbit needing to interpret a raptor attack in order to escape it. Under the best possible scenario, it may take ~ 100 ms or more for the rabbit to perceive the attack, and even longer to combine these events with short term and long term memory (Madl et al., 2011). Based on the evolutionary process, this specific time window is sufficient to develop a successful escape strategy while optimizing the finite resources of its brain and body, considering the natural environment, in which the rabbit's ancestors strived for millions of years.

In this work, we outlined a framework for interpreting and modeling brain measurements demonstrating metastable dynamics with rapid transients, which can be used to develop computational devices incorporating brain-inspired principles. Such novel devices have the potential to develop machines which understand the world around us in a way as we humans do, and help us with the challenges we face.

## Data Availability Statement

The data analyzed in this study is subject to the following licenses/restrictions: data are available upon request from the authors. Requests to access these datasets should be directed to rkozma@memphis.edu.

## Author Contributions

RK coordinated the work with a focus on mathematical and computational modeling and outlined the initial draft manuscript that was modified, and approved by all authors in its final version. BB and NG focused on consciousness and cognitive areas. All authors contributed to the conceptual formulation of this research.

## Conflict of Interest

NG founded MedNeuro, Inc. The remaining authors declare that the research was conducted in the absence of any commercial or financial relationships that could be construed as a potential conflict of interest.

## Publisher's Note

All claims expressed in this article are solely those of the authors and do not necessarily represent those of their affiliated organizations, or those of the publisher, the editors and the reviewers. Any product that may be evaluated in this article, or claim that may be made by its manufacturer, is not guaranteed or endorsed by the publisher.

## Footnotes

^1^In the scientific literature, over 27 thousand relevant abstracts can be found at this link: https://pubmed.ncbi.nlm.nih.gov/?term=conscious+brain.

^2^Stan Franklin's research group really pushed the world of computer science and AI toward these cognitive architectures and moved the needle into this direction, in a 20+ year strong research program at the University of Memphis.

^3^We take no position on philosophical questions that are often raised in connection with conscious perception, the brain, and the relevance of quantum mechanics and quantum fields. Global workspace theory and neuropercolation should be considered on their respective merits. Both theories have been fruitful, and here we consider how they may interact in interpreting experimental results.

^4^A state of a dynamical system is called metastable, if it is not stable, but it maintains its integrity for an extended period of time, which is meaningful for the analyzed problem. In other words, a metastable state is unstable over very long time scales, but it can be considered stable for shorter, still extended time periods. It is of special interest to study metastability in spatially extended systems, when metastability in time is manifested in the emergence of well-defined spatial patterns for some time periods. Transient dynamics from one metastable state to another metastable state has been extensively studied in various mathematical and physical systems. In the present essay, we refer to metastability appearing in the form of intermittent synchronization of cortical activity.
